# Medical Society of the County of Albany

**Published:** 1872-04

**Authors:** James S. Bailey


ART. II—*Medical Society of the County of Albany. Semi-Monthly Meeting, March 20th,* 1872. *Reported by* JAMES S. BAILEY, M. D.

Dr. JOSEPH LEWI, President, in the Chair.

Dr. THOMAS BECKETT, remarked upon *Cerebro-Spinal Meningitis,*

and referred to the disease as occurring as an epidemic in the western part of the city within the boundary of his practice.

The symptoms were characterized by extreme headache, and pain in the back of the neck, the patients being unable to bend their necks forward, with constipation and an eruption similar to measles.

Recovery ordinarily took place in the course of four weeks.

Dr. B. detailed several cases, as follows:

Case 1. A young man aged 19, a drummer, turned out on the 22d of February, and took cold, was taken sick the next day; a slight eruption appeared over his bowels, tongue coated, and epithelial deposits over fauces, complained of headache, and was delirious and quite deaf. Gave Bromide of ammonium, and the patient recovered.

Case 2. Complained of pain in the back of the head, which extended half way down the spine, pupils contracted, and coldness of extremities; was insensible, gave aromatic spts. of ammonia with warm mustard baths; patient recovered. Other cases were mentioned but did not present any peculiar symptoms.

Dr. Lorenzo Hale, also reported some cases of *Cerebro-Spinal Meningitis,* recently occurring in his practice, with not the same favorable results as Dr. Beckett. About six weeks ago he was called to see two cases in the same family, a boy aged 6 and a girl aged 3 years. They were taken with vomiting and chilliness and pain in the head, bowels constipated; upon the third day an eruption appeared. The girl had no rash, opisthotonos was present in both cases. The oldest case could not hear after the first week; after the second week they were considered convalescent, and the Dr. was discharged by the parents; in less than a week, the Dr. was summonsed in haste; the boy died that night, the girl recovered.

The third case, was attacked with decided trismus which lasted 48 hours; there was no apparent change in the pupil; pulse not more than 90, herpes scattered over the face; no loss of consciousness.

Dr. A. Vanderveer, mentioned a case he had seen with Dr. O'Leary. The boy was 17 years old, was skating on the river, became fatigued and laid down upon the ice and slept a little, he awakened chilly. The next day went to school, vomited and be came delirious. There was tenderness along the spine.

Treatment, Bromide of potash with cathartics, gave chloral to make him rest and nourished him well, opisthotonos was well marked until the 29th day, but very little delirium after the third week. Quinine and pyrophsphate ferri was then given with iodide potash ; there was no eruption, the patient will recover.

Dr. Porter inquired whether the eruption was general ? and remarked it had not been in the few cases he had seen.

Dr. Estes H. Davis, related two cases of cerebro-spinal meningitis illustrating extremes. Case 1. A lad 10 years of age attended a protracted meeting at 4 P. M., was seemingly in good health, was taken sick in church, and when he came home complained of feeling cold, and had a singular wild expression. Dr. D. was called soon after the attack, called again at 6 o'clock, he was still cold, and his skin looked dusky, was delirious with quick pulse; his delirium soon amounted to frenzy; nothing relieved him; he would stand upon the bed, scream and if not restrained, dive off without regard to personal safety; there was no convulsions. He lived only 9 hours.

Case 2. A young lady 18 years of age, went to bed apparently in perfect health, she awakened in the night and screamed which soon approached a wild frenzy; her head was slightly drawn backward, her eyes had a glairy wild expression, both cases were covered with dark patches of eruptions, her eyes were drawn directly inward, pupils were contracted, skin cold. The delirium continued until 9 A. M., then re-action took place and she was feverish. She was delirious a part of the time for four days, her head was drawn backward nearly to the spine; the Dr. without any authority except his best judgment, bled her moderately, the fourth day after seizure, with decided relief. This was done as a feeler, the blood was heavily buffed and cupped. The next day he took less than a pint of blood, which seemed to improve her, she soon became conscious, her eyes gradually returned to their normal position, her arms were then drawn up, and she had difficulty in breathing and of retaining anything upon her stomach, her limbs drew up close to her body, and she could not endure the pain in having them straightened. In the course of four weeks the discoleration improved, and she was able to go around at the expiration of four months.

Dr. JAMES S. BAILEY reported a case of cerebro-spinal meningitis,that occurred in his practice about six weeks ago;there was present the usual pain in the head and neck, intolerance to light and delerium, opisthotonos, etc. ; treatment, emplastrum cantharides, 2x5 over the spine extending from the neck down, bromide of potash, with diaphoretics alternately, nourished well and kept the bowels open, several physicians saw the case and confirmed his diagnosis.

Just fourteen days from the attack another child in the same family was taken with scarlet fever, and in three days more another was taken. Dr. B. would have mistrusted its having been a case of scarlet fever, although there were involuntary discharges from bowels and bladder, and the patient was left with strabismus, had she not have taken scarlet fever also at the expiration of the 18th day.

Dr. Bailey remarked, as the subject of scarlet fever had been mentioned, he would give some interesting cases recently occurring in his practice.

He was called to see a child who had died suddenly without apparent cause. She had complained slightly for a day or two previously, but went to bed seemingly in usual health; towards morning she called her father to get her a drink of water, when it was brought she was dead. Before he left the house, a grown young man consulted him in reference to himself; upon exposing his breast he found that he was scarlet with the rash. In due time every member of the family were attacked with scarlet fever. A post mortem in the case of the little girl, revealed the fact that she died from scarlet fever.

Dr. B. continued, not long since I was called to see a little girl about four years old, she had a violent fever, breathed short and rapid, with some interruption and a slight cough. The nostrils were peculiarly expanded, indicating irritation or inflammation of the air passages; in short her symptoms resembled an acute case of bronchitis.

The symptoms became so urgent that he invited several physicians to see the case with him. Not one of them suspected the true cause of the sickness, until about the 10th or 11th day from the attack, when a purplish eruption began to make its appearance about the face and neck, which was shortly developed into a thorough rash as is usual in cases of this fever. Then the alarming symptoms subsided, and she made a good though slow recovery. There were two more children in the same family, one of them a new born infant, both escaped.

I am satisfied, Mr. President, we frequently lose cases of congestive scarlet fever, when the rash does not appear, and the cause of death is never known. I am at the present time attending a little boy who had a slight rash seven days before I saw him. The immediate cause of alarm was the induration that appeared under the left ear. At the expiration of three days I penetrated it deep with my lancet and a quantity of pus escaped; every few days abscesses formed in various parts of the body, and pus accumulated in them; in the short space of three days after,inflammation manifested itself. After the lad had been sick about one month purpura hemorrhagia appeared, and several of the incised abscesses bled freely, which were arrested by the stiptic application of ferri per sulphas applied by means of pledgets of lint to the bleeding surface.

He has now been sick nearly seven weeks, and has nine open abscesses besides others forming.

Scarlet fever has appeared in my practice under such a variety of circumstances, that I have often doubted its infectiousness. I have seen it prevail in rural districts, in families living as it were isolated, and several miles from any other habitation, and without any known exposure. Even when the members of the family had not been away from home in a month, nor had they received a visitor during that time.

When other cases appeared they usually occurred about the 14th day after the first.

I have frequently attended one case in a family when every other member was exposed but did not contract it. I have also attended other families when every one of their number had the fever.

I have concluded that persons living remote from habitations are as much exposed to scarlet fever, as persons being in crowded cities.

Four years ago, about Christmas, one of my children was taken with scarlet fever, while three other children escaped, although they were demonstrative in their affection for the sick one, kissing her repeatedly and sitting for hours by her bed-side; yet strange to say another case did not appear until twelve months more had transpired, when I had another case, to be followed by another at an interval of another year, before the third case appeared.

Those of my friends who knew the circumstances, were wondering whether my fourth child would contract the scarlet fever at the expiration of another year. Sure enough this really was the case. To say the least this was a singular coincidence.

